# Impact of Fruit and Vegetable Protein vs. Milk Protein on Metabolic Control of Children with Phenylketonuria: A Randomized Crossover Controlled Trial

**DOI:** 10.3390/nu14204268

**Published:** 2022-10-13

**Authors:** Alex Pinto, Anne Daly, Júlio César Rocha, Catherine Ashmore, Sharon Evans, Richard Jackson, Anne Payne, Mary Hickson, Anita MacDonald

**Affiliations:** 1 Dietetic Department, Birmingham Women’s and Children’s Hospital, Birmingham B4 6NH, UK; 2 Faculty of Health, Plymouth Institute of Health and Care Research, University of Plymouth, Plymouth PL6 8BH, UK; 3 NOVA Medical School|Faculdade de Ciências Médicas, NMS|FCM, Universidade Nova de Lisboa, 1169-056 Lisboa, Portugal; 4 CINTESIS, NOVA Medical School|Faculdade de Ciências Médicas, NMS|FCM, Universidade Nova de Lisboa, 1169-056 Lisboa, Portugal; 5 Reference Centre of Inherited Metabolic Diseases, Centro Hospitalar Universitario de Lisboa Central, 1169-045 Lisboa, Portugal; 6 Cancer Research UK Liverpool Cancer Trials Unit, University of Liverpool, Liverpool L69 3GL, UK

**Keywords:** phenylketonuria, fruits, vegetables, milk protein, metabolic control, phenylalanine

## Abstract

Fruits and vegetables containing phenylalanine ≤ 75 mg/100 g (except potatoes) have little impact on blood phenylalanine in phenylketonuria (PKU). In a randomized, controlled, crossover intervention trial, we examined the effect of increasing phenylalanine intake from fruits and vegetables, containing phenylalanine 76–100 mg /100 g, compared with milk protein sources on blood phenylalanine control. This was a five-phase study (4 weeks each phase). In Phase A, patients remained on their usual diet and then were randomly allocated to start Phase B and C (an additional phenylalanine intake of 50 mg/day, then 100 mg from fruits and vegetables containing phenylalanine 76–100 mg/100 g) or Phase D and E (an additional phenylalanine intake of 50 mg/day then 100 mg/day from milk sources). There was a 7-day washout with the usual phenylalanine-restricted diet between Phase B/C and D/E. Blood phenylalanine was measured on the last 3 days of each week. If four out of six consecutive blood phenylalanine levels were >360 μmol/L in one arm, this intervention was stopped. Sixteen patients (median age 10.5 y; range 6–12 y) were recruited. At baseline, a median of 6 g/day (range: 3–25) natural protein and 60 g/day (range: 60–80) protein equivalent from protein substitute were prescribed. Median phenylalanine levels were: Phase A—240 μmol/L; Phase B—260 μmol/L; Phase C—280 μmol/L; Phase D—270 μmol/L and Phase E—280 μmol/L. All patients tolerated an extra 50 mg/day of phenylalanine from fruit and vegetables, containing phenylalanine 76–100 mg/100 g, but only 11/16 (69%) tolerated an additional 100 mg /day. With milk protein, only 8/16 (50%) tolerated an extra 50 mg/day and only 5/16 (31%) tolerated an additional 100 mg/day of phenylalanine. Tolerance was defined as maintaining consistent blood phenylalanine levels < 360 μmol/L throughout each study arm. There was a trend that vegetable protein had less impact on blood phenylalanine control than milk protein, but overall, the differences were not statistically significant (*p* = 0.152). This evidence supports the PKU European Guidelines cutoff that fruit and vegetables containing 76–100 mg phenylalanine/100 g should be calculated as part of the phenylalanine exchange system. Tolerance of the ‘free use’ of these fruits and vegetables depends on inter-patient variability but cannot be recommended for all patients with PKU.

## 1. Introduction

Phenylketonuria (PKU) (PKU, OMIM—Online Mendelian Inheritance in Man # 261,600) is a rare inherited metabolic disorder caused by mutations in the phenylalanine hydroxylase gene leading to phenylalanine hydroxylase deficiency and an inability to convert phenylalanine into tyrosine [1]. High blood and brain phenylalanine causes irreversible intellectual disability and developmental, behavioral and psychiatric difficulties [1]. The primary treatment of PKU is a phenylalanine-restricted diet. It consists of three main principles: (1) exclusion of high phenylalanine foods, e.g., meat, fish, eggs, cheese, bread, flour, pasta, nuts, seeds, and aspartame; (2) provision of phenylalanine requirements from weighed amounts of foods such as potatoes, spinach, and peas, and (3) administration of a synthetic protein substitute that is usually supplemented with vitamins and minerals that provides up to 80% of protein requirements [2].

The protein content of fruits and vegetables varies between 0.3–2.7 g and 0.5–6.0 g per 100 g, respectively. The minimum and maximum phenylalanine/protein ratios (smallest and highest amount of phenylalanine per 1 g of protein, respectively) have been established: fruits contain 20–39 mg phenylalanine per 1 g of protein; vegetables contain 20–40 mg phenylalanine per 1 g of protein (except spinach, peas, seaweed, kale, and sweetcorn which have a higher ratio) [3]. In PKU, UK expert opinion concerning the suitability of fruits and vegetables in a phenylalanine-restrictive diet has changed with time. Since the 1970s, fruits and vegetables containing phenylalanine ≤ 50 mg/100 g of food were permitted without measurement or restriction. Prior to this, 20 mg exchange amounts of fruits and vegetables were calculated in the diet [4]. In 2003, fruits and vegetables containing ≤ 75 mg of phenylalanine/100 g were given without measurement in the Birmingham (UK) PKU clinic [5].

There is both short and long-term evidence from three studies in different PKU international centers to suggest that the consumption of fruits and vegetables containing phenylalanine ≤ 75 mg/100 g without measurement or restriction is not associated with elevated blood phenylalanine [5,6,7,8]. Therefore, the European PKU guidelines 2017 recommended that fruits and vegetables (except potatoes) containing phenylalanine up to 75 mg/100 g could be given without restriction [9]. There is also some evidence in children to suggest that vegetables containing phenylalanine between 76 and 100 mg/100 g can be given without measurement but in small portions only [5]. In Austria, up to five portions a day (one portion defined as a handful) of fruits and vegetables containing phenylalanine up to 100 mg/100 g (excluding potatoes) were given without adverse effect on control [8].

It is uncertain why fruits and vegetables containing phenylalanine ≤ 75 mg/100 g have limited impact on metabolic control in PKU. The digestibility of protein in foods is dependent on many factors including their food matrix and microstructure, protein amino acid profile, protein folding and crosslinking leading to variation between plant and animal protein [10]. It is also probable that the digestion and absorption kinetics of proteins differs in fruits and vegetables, and this possibly affects post-prandial phenylalanine concentrations [11,12]. Protein digestibility corrected amino acid scores (PDCAAS) is a composite indicator of protein quality that has been used to measure the ability of protein from different food sources to meet amino acid requirements [13]. PDCAAS have a value from 0 to 1, and it has been estimated that milk and eggs have a value of 1 [12] compared with 0.73 for vegetables, 0.70 for legumes and 0.64 for fresh fruits [14]. Some authors suggest that the digestible indispensable amino acid score (DIAAS) is a better method to evaluate protein quality, as it defines amino acids as individual nutrients, and that protein quality is contingent on indispensable amino acid content and ileal (as opposed to fecal) digestibility. These scores do define animal protein as more digestible compared with vegetable protein, but they are not widely available [15,16].

In PKU, the overall evidence that unmeasured intake of fruits and vegetables containing phenylalanine between 76 and 100 mg/100 g does not adversely affect blood phenylalanine is limited and uncertain. It is also unclear if patients with PKU can tolerate a higher daily amount of phenylalanine from plants compared with animal sources. We aimed to examine in a randomized controlled crossover intervention trial the effect of increasing phenylalanine intake from fruits and vegetables containing phenylalanine from 76 to 100 mg/100 g compared with equivalent phenylalanine from milk protein sources on fasting blood phenylalanine levels in children with PKU.

## 2. Materials and Methods

### 2.1. Patient Selection

Patients eligible for this study were children with PKU, aged 5 to 12 years managed at Birmingham Women’s and Children’s Hospital Trust, UK. Participants were diagnosed with PKU by newborn screening and early treated by a phenylalanine-restricted diet supplemented with a low/free phenylalanine protein substitute. Patients were in good general health as evidenced by their medical history, had no co-morbidities that could affect blood phenylalanine control and were able to comply with the study protocol. Three of four consecutive blood phenylalanine levels were <360 μmol/L immediately before study commencement (if two out of four were above the target range but close to 360 μmol/L, participation was determined by the stability of metabolic control in the previous 6 months, i.e., the majority of blood phenylalanine levels had to be <360 μmol/L in the previous 6 months).

Exclusion criteria were co-morbidities affecting blood phenylalanine control (e.g., diabetes, renal or gut disorders), dislike of fruits and vegetables containing phenylalanine from 76 to 100 mg/100 g (e.g., broccoli, cauliflower) or milk products (e.g., yoghurt), sapropterin dihydrochloride use, children with biopterin defects, and the presence of intercurrent infection at the time of study commencement.

### 2.2. Study Design

This was a single-center randomized controlled crossover study to compare the effects of increasing phenylalanine intake from different sources (milk vs. fruit and vegetable protein) on blood phenylalanine. The study comprised five phases (4 weeks each with one week washout between B/C and D/E) over 21 weeks:

- **Phase A**: no change to dietary intake.
- **Phase B and C**: usual diet with an additional 50 mg and 100 mg/day of phenylalanine from fruits and vegetables containing phenylalanine 76–100 mg/100 g, respectively.
- **Phase D and E**: usual diet with an additional 50 mg and 100 mg/day of phenylalanine from milk protein sources, respectively.

Patients were randomized using a 1:1 ratio between the following two treatment groups: Group 1 (Phase A, B, C, D, E) and Group 2 (Phase A, D, E, B, C).

**Figure 1:**
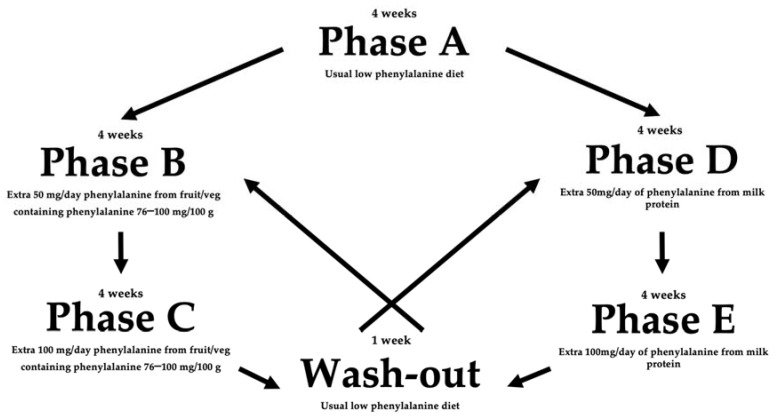
Randomized controlled crossover study design.

On the last 3 days of each week (commonly Friday, Saturday, Sunday), caregivers took morning fasting blood spots for phenylalanine from their children and recorded 3-day food diaries. There was a 7-day washout period when patients returned to their usual diet between each of the two-part, four-week intervention periods. If four of six consecutive blood phenylalanine levels were >360 μmol/L in one arm of the study, this phase of the study was stopped. If this occurred in Phase B or D of Group 1 and 2, respectively, patients would move to the other intervention (Phase C or E) after the washout period. A detailed study design is presented in Figure 1.

The phenylalanine content (mg/100 g) and the portion sizes given for a 50 mg phenylalanine exchange for all the fruit/vegetable and milk protein exchanges given in the intervention period are listed in Table 1.

### 2.3. Data Collection

Data were collected between 29 July 2019 and 11 January 2021. Anthropometric measurements (weight and height) were performed by trained dietitians (A.M. and A.P.) at baseline (Phase A) and at the end of the study, and weight was recorded on day one of each study phase. Height was measured using a Seca stadiometer to the nearest 0.1 cm and weight on calibrated digital scales to the nearest 0.1 g (Seca, Medical Measuring Systems and Scales, Birmingham, UK—Model 875).

Any fruit and vegetables eaten as part of the intervention were recorded daily by parents throughout the study. A weighed diet diary was completed by parents/caregivers for the last three days of each week on the same days as additional blood spots were taken for phenylalanine. This was checked by AM and AP. Protein substitute amount, type, dose and timing was also recorded. Energy and macronutrient intake were analyzed with Nutritics® (v5.09*, Dublin, Ireland). The protein equivalent intake (g/day and g/kg) from protein substitute and protein intake from foods (g/day) was calculated for each phase of the study.

Trained parents/caregivers collected morning fasting blood spots on the last three days of each week on filter cards, (Perkin Elmer 226, Waltham, MA, USA). At least two blood spots on each filter card were collected, and blood phenylalanine was calculated on a 3.2 mm punch by MS/MS tandem mass spectrometry.

### 2.4. Statistical Analysis

The primary outcome measure of this study was the mean weekly fasting blood phenylalanine from three daily blood spots taken each week over 20 weeks. The secondary outcomes were dietary intake based on 3-day diet diary, protein substitute and natural protein intake and natural protein amounts from each food source to compare how different protein sources (animal or vegetable protein) influence blood phenylalanine control in PKU. For a study with a two-sided alpha level of 0.05, a total of 13 patients were required to obtain a statistical power of 90%. We aimed to recruit 16 patients to account for any dropouts. All the statistical analysis was performed with R (version 3, R Foundation for Statistical Computing, Vienna, Austria). Statistical significance was determined by a *p*-value < 0.05, and all results were reported alongside 95% confidence intervals. Continuous data were summarized as median (IQR) or mean (SD) depending on normality testing, whilst categorical data were summarized as number of times with associated percentages. Assessment of the change in outcome data over time were evaluated using Analysis of Covariance (ANCOVA), which was adjusted for baseline covariates. Models for phenylalanine as the primary endpoint were performed including baseline phenylalanine as a covariate along with terms which adjust for study period, weeks and randomization groups.

### 2.5. Ethical Aspects

The study was conducted in full conformance with the principles of the “Declaration of Helsinki” (52nd WMA General Assembly, Edinburgh, Scotland, 3–7 October 2000) and Good Clinical Practice guidelines. Ethical approval was given by the East Midlands—Leicester South Research Ethics Committee with the references 19/EM/0073 and Integrated Research Application System (IRAS) number 252561. This project is also registered in clinicaltrials.gov with the ID: NCT05249218.

Informed consent was obtained prior to data collection from parents/caregivers and age-appropriate assent from participating patients.

## 3. Results

### 3.1. Participants

Sixteen children with PKU (69% females, *n* = 11/16) were considered eligible and agreed to participate in this study. The median age was 10.5 years (range: 6–12). Most children had classical PKU (*n* = 14), and two had mild PKU based on their blood phenylalanine at the time of diagnosis and phenylalanine tolerance (with only two children able to tolerate ≥10 g/day natural protein at the beginning of the study). Fourteen children were European and two were of Asian origin. All patients were able to eat the necessary portion sizes of the fruits and vegetables or milk products required by the study intervention.

At the start of the study, patients were prescribed a median of 6 g/day (range 3–25) natural protein and a median of 60 g/day (60–80) of protein equivalent from protein substitute. Individual patient’s characteristics and dietary prescriptions at the start of the study are described in Table 2. Patients were randomized to groups 1 or 2 on recruitment (randomization did not affect results using a linear model which included baseline blood phenylalanine levels as an adjusting covariate, *p* = 0.945).

### 3.2. Intervention

In total, 16 patients completed 4 weeks of Phase B (one extra 50 mg/day phenylalanine exchange), 14 patients completed 4 weeks of Phase C (two extra 50 mg/day phenylalanine exchanges), 11 patients completed 4 weeks of Phase D (one extra 50 mg/day phenylalanine exchange) and 6 patients completed 4 weeks of Phase E (two extra 50 mg/day phenylalanine exchanges) intervention.

### 3.3. Tolerance of Additional Phenylalanine Intake

All patients (*n* = 16) were able to tolerate an extra 50 mg/day of phenylalanine from fruits and vegetables containing phenylalanine 76–100 mg/100 g, but only 69% (*n* = 11/16) tolerated an extra 100 mg/day from this source. With milk protein, only 50% of patients (*n* = 8/16) tolerated an extra 50 mg/day of phenylalanine and 31% (*n* = 5/16) an extra 100 mg/day (Table 3). Tolerance was defined as maintaining consistent blood phenylalanine levels < 360 μmol/L throughout each study arm. Either intervention was stopped if blood phenylalanine increased above the upper target range for four of six consecutive blood levels. The length of time taken for phenylalanine levels to elevate above the upper target range in response to extra dietary phenylalanine was variable for each patient (Table 4).

### 3.4. Impact on Blood Phenylalanine Control and Differences between Milk Protein vs. Fruit/Vegetable Protein

**Figure 2:**
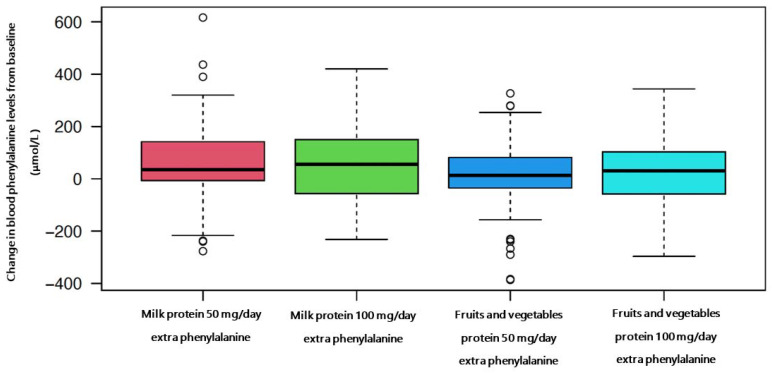
Mean change of blood phenylalanine from baseline in each intervention over 4 weeks. Mean blood phenylalanine (range) from baseline: 50 mg/day of phenylalanine from milk protein (35 μmol/L; −7, 142) *n* = 8; 100 mg/day of phenylalanine from milk protein (56 μmol/L; −56, 149) *n* = 5; 50 mg/day of phenylalanine from fruits and vegetables (13 μmol/L; −34, 79) *n* = 16; 100 mg/day of phenylalanine from fruits and vegetables (30 μmol/L; −58, 103) *n* = 13. The circles represent outliers.

Median blood phenylalanine levels in Phase A were 240 μmol/L (from *n* = 183 blood spot samples), Phase B: 260 μmol/L (*n* = 182 blood spot samples), Phase C: 280 μmol/L (*n* = 170 blood spot samples), Phase D: 270 μmol/L (*n* = 155 blood spot samples) and Phase E: 280 μmol/L (*n* = 81 blood spot samples). The mean change from baseline levels is presented in Figure 2.

The model showed that the difference between the two interventions was a phenylalanine level of −24 μmol/L for the fruits and vegetables intervention compared to milk protein, although this did not reach statistical significance (*p* = 0.152). The difference in mean blood phenylalanine levels when giving an extra 50 mg/day of phenylalanine from fruit and vegetables vs. milk protein was −35 μmol/L (±21; *p* = 0.107) and with an extra 100 mg/day of phenylalanine was −8 μmol/L (±26; *p* = 0.768).

**Figure 3:**
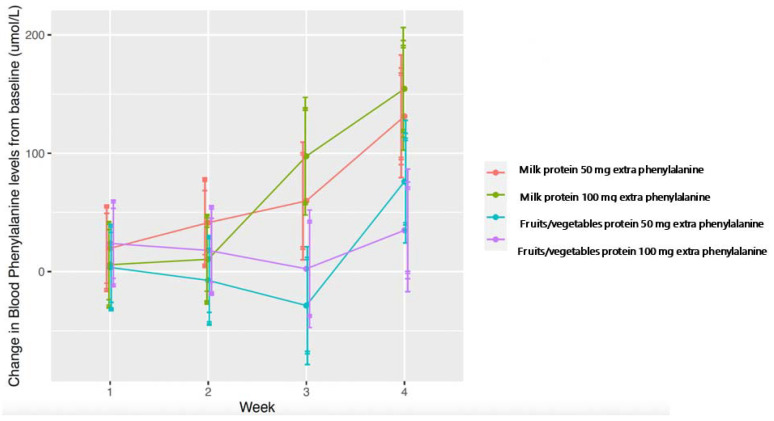
Mean weekly change in blood phenylalanine from baseline in the fruits and vegetables and milk protein intervention.

**Figure 4:**
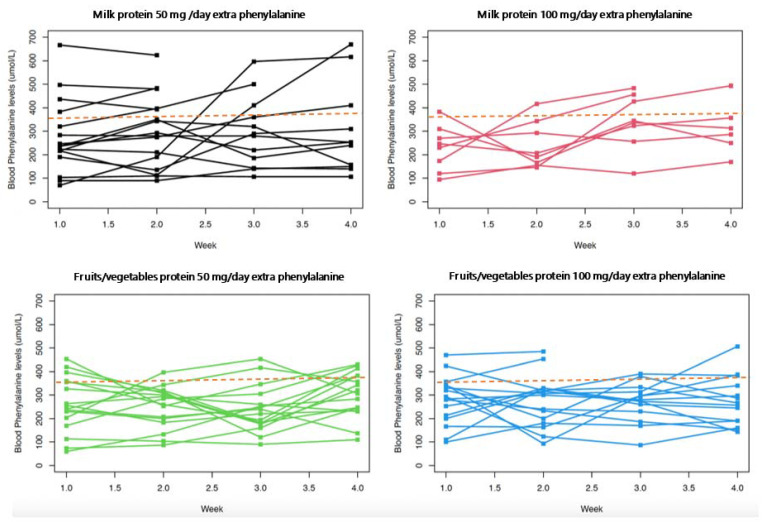
Individual blood phenylalanine levels over 4 weeks with each intervention. Dotted line shows recommended maximum blood phenylalanine concentration (360 μmol/L). Black lines represent blood phenylalanine levels of all individual patients with 50 mg/day of extra phenylalanine from milk protein. Red lines show blood phenylalanine levels of all individual patients with 100 mg/day of extra phenylalanine from milk protein. Green lines represent blood phenylalanine levels of all individual patients with 50 mg/day of extra phenylalanine from fruit/vegetable protein. Blue lines show blood phenylalanine levels of all individual patients with 100 mg/day of extra phenylalanine from fruit/vegetable protein.

Figure 3 represents changes in mean blood phenylalanine from baseline over each four-week phase, and Figure 4 shows the changes over each week of the intervention period. A higher and faster increase in blood phenylalanine is observed with milk protein intervention. A more prolonged response is observed with fruit and vegetable protein. Responses may have been even more pronounced if the intervention had not been stopped when blood phenylalanine exceeded the target range. In Figure 4, individual blood phenylalanine levels are given for each week of all phases B to E. There is less stability of blood phenylalanine demonstrated by its higher and greater variability with extra milk protein compared with fruits and vegetables.

The mean change in blood phenylalanine was not statistically significant during the fruits and vegetables intervention. This was similar for each week of the milk protein intervention until week 4 of an additional 100 mg/day phenylalanine when there was a mean increase from baseline of 154 µmol/L (*p* = 0.049). A clinically relevant change in blood phenylalanine (76 µmol/L) was observed in week 4 when 50 mg/day of phenylalanine from fruits and vegetables was given compared with a mean increase of 41 µmol/L from week 2 of 50 mg/day of phenylalanine of milk protein (Table 5).

### 3.5. Impact on Phenylalanine Intake

Any change in phenylalanine intake in each study phase is given in Table 6. These changes should be interpreted carefully, as only in Phase A and B did all 16 patients complete the full 4 weeks of intervention. In Phase C, D and E only 14, 11 and 6 patients fully completed the 4 weeks intervention due to loss of blood phenylalanine control in the other subjects.

The main food eaten during Phase B and C was broccoli. In Phase D and E (milk protein), the extra phenylalanine was provided by milk, yoghurt or milk-based ice cream. The foods used to provide extra phenylalanine and the number of times they were used during the 3-day diet diary assessment are reported in the Supplementary Table S1.

### 3.6. Nutritional Intake

Two hundred and seventy-five 3-day dietary assessments were analyzed with a median of seventeen per subject. The dietary intake analysis is presented in Supplementary Table S2. There was no significant change in energy intake throughout the study between milk protein compared with fruit and vegetables intervention (*p* = 0.188).

Throughout the study, the overall median daily energy intake was 1669 Kcal/day (range 1578–1770), with a median percentage of estimated average requirement of 85% (79–89%). The median carbohydrate intake was 224 g/day (range 206–238), providing 54% (range 52–56) of the energy intake; median fat intake was 52 g/day (range 44–56 g) contributing 28% (range 26–39%) of the energy intake, and the median total protein intake (protein substitute and natural protein) was 75 g/day (71–80 g) providing 18% (range 17–20) of energy intake.

### 3.7. Anthropometry

The median height z-score at baseline was 1.04 (range: 0.29–2.01) and 1.20 (range: 0.44–2.12) at the end of the study.

The patient median weight z-score at baseline was 1.34 (range 0.78–2.51) and 1.65 (range 0.13–2.96) at the end of the study. In Phase B, the median weight z-score was 1.44 (range 0.88–2.58; *n* = 16), while in Phase C, it was 1.46 (range 0.99–2.61), in Phase D, it was 1.67 (range 1.13–2.92; *n* = 16), and in Phase E, it was 1.51 (range 1.22–1.74; *n* = 8).

## 4. Discussion

This randomized, controlled trial is the first study to show that the unlimited use of fruits and vegetables containing phenylalanine 76–100 mg/100 g was not tolerated by children with PKU on dietary treatment only. Although all children were able to tolerate an extra 50 mg/day phenylalanine from these fruits and vegetables, they could not tolerate 100 mg/day, with over 50% of children increasing their blood phenylalanine levels above the European PKU guideline therapeutic target range [9]. While these results are disappointing, they suggest that the European PKU guideline statement that specifies that the intake of all fruits and vegetables that contain phenylalanine > 76 mg/100 g should be controlled and measured within the daily phenylalanine allocation is appropriate.

Understanding the upper phenylalanine content of fruits and vegetables that can be tolerated without measurement in patients with PKU on dietary treatment only is particularly important. In PKU, the rate of obesity although similar to the general population is high [18], but fruit and vegetable intake is low [19]. It is well established that vegetables are good sources of bioactive compounds, including dietary fiber, micronutrients and phytochemicals, and they are associated with a reduced risk of chronic disease; strategies to increase the intake and variety of low-energy-dense vegetables are essential [20]. However, vegetable consumption that can only be eaten in controlled and limited amounts may be minimal in PKU, as patients may prefer to use their phenylalanine allocation on milk, cereals, and potato products instead. Unfortunately, many vegetables such as broccoli, spinach, chard, mange tout, sugar snap peas, and bean sprouts contain phenylalanine ≥ 76 mg/100 g, so their phenylalanine content should continue to be calculated and measured. Vegetable rice made from cauliflower and broccoli or steaks made from cauliflower are popular in the general population and would have increased meal choice, but their intake will remain constrained in patients with PKU treated by diet only. In addition, the control of fruit and vegetable consumption may limit even further the intake of fiber, possibly contributing to dysbiosis and increasing the risk of metabolic comorbidities.

Our results did suggest that patients (*n* = 16) were able to tolerate 50 mg/day of phenylalanine from fruits and vegetables containing phenylalanine 76–100 mg/100 g but not more than this. These results were similar to an earlier study that showed that fruits and vegetables with a phenylalanine content between 76 and 100 mg/100 g could be given without measurement for 7 weeks but only because the portion size was small and provided a mean of 39 mg/day of phenylalanine only. This amount of phenylalanine from the very limited intake of these vegetables had a minimal impact on blood phenylalanine levels [5], but the authors recommended caution and that further research was necessary before recommendations could be changed.

Our results suggested a trend that phenylalanine from plant protein may be better tolerated than milk protein, although the results were not statistically significant. Milk protein was associated with an immediate and sharper rise and less stability of blood phenylalanine. However, not all patients were tested over 4 weeks with extra phenylalanine from milk protein due to loss of metabolic control, so the differences we observed could have been greater. Milk protein has a high digestibility estimated at 95% in the ileum [21]. It is present within a complex matrix that affects digestive and absorption processes [22]. Milk protein is comprised primarily of whey and casein proteins, which constitute around 20 and 80% of the total protein fraction, respectively [12]. The kinetics of protein digestion both within milk protein (casein vs. whey) and plant protein differs. Casein protein is associated with a slow delivery of amino acids to the systemic system, and whey protein is associated with a slower rate of amino acids in the peripheral circulation when compared with soya proteins [23]. In contrast, whey protein contains a high amount of ß-lactoglobulin which transits more rapidly to the upper jejunum than casein, and it consequently undergoes more rapid digestion. This is primarily due to the solubility of ß -lactoglobulin in the acid environment of the stomach resisting gastric digestion [21]. Whey protein contains 10 to 11% leucine compared with 8% in casein and only 6–8% in plant protein [24]. The role of leucine in stimulating an anabolic response in skeletal muscle and anabolic recovery is well documented, although the overall amount of leucine provided from milk protein was small in this study [10,25].

It is well established that plant protein is less digestible (only 50–80%) compared with milk protein (milk digestibility score is 0.96 when assessing from 0–1) [10]. The secondary structure of plant proteins is characterized by a high content in β-sheet conformation and a relatively low α-helix amount compared to animal proteins. The high content in β-sheet conformation may be connected to its resistance to proteolysis in the gastrointestinal tract. Hence, the hydrophobic β-sheet structure of plant proteins that facilitates protein aggregation causes decreased digestibility. Plant-based proteins also contain non-starch polysaccharides or fiber in cell walls that impede the access of enzymes to proteins and could reduce protein digestibility. The presence of some anti-nutritional factors, such as phytic acid, protease inhibitors, hemagglutinins, glucosinolates, tannins, and gossypol, could also affect the digestibility of plant-based protein sources and increase losses of endogenous protein at the terminal ileum. Food processing and heat will also alter plant protein digestibility [10,26,27,28]. These factors may explain why fruits and vegetables containing phenylalanine ≤ 75 mg/100 g can be given without measurement in a phenylalanine-restricted diet, even though they may provide an additional 20% of phenylalanine intake.

Surprisingly, two patients in our cohort were able to tolerate 100 mg/day phenylalanine from animal protein but not plant protein. This is difficult to explain but could be related to the presence of other food compounds within the diet that influenced peptidase degradation and the intestinal transport of both plant and milk protein. Fat slows gastric emptying, and the presence of lipid products within the duodenum induces the secretion of biliary and pancreatic fluids that impact on absorption. Differences because of individual protein tolerances, gene–diet interactions, gut microbiota or the oral mastication process could influence the physiological outcomes on phenylalanine bioavailability [21,29,30]. In this study, children ate similar meals from week to week, but other foods consumed may have altered milk and plant protein availability.

When taking plant protein, all children were able to tolerate an extra 50 mg/day phenylalanine but only 50% (*n* = 8/16) when given an additional 50 mg/day phenylalanine from animal protein. Some of this may be explained by patients being prescribed less phenylalanine than their maximum tolerance, which is similar to a Portuguese study [31], but in our cohort of well-controlled children with PKU, most of the children could not tolerate an additional 100 mg/day of phenylalanine without loss of metabolic control. In this study, almost all patients had classical PKU, and they were periodically tested with additional dietary phenylalanine. We observed interpatient variation in the length of time before patients experienced an elevation in blood phenylalanine in response to the challenge with additional phenylalanine intake. Some patients increased blood phenylalanine within 14 days, but some taking up to 28 days with each intervention. Although it is important to optimize phenylalanine tolerance, this study highlighted some of the practical difficulties in achieving this. Most parents choose to maintain their child’s blood phenylalanine levels comfortably below the European PKU Guidelines upper therapeutic target level [9].

Multiple factors influence metabolic control in PKU. Energy intake, catabolic state caused by illness or low energy intake, dose and timing of protein substitute intake blood spot timing (fasting/non fasting state), and quality of blood spots for phenylalanine analysis are all important. In this study, blood spots were all taken fasting, pre-breakfast and at the same time each day. The timing and dose of protein substitute was standardized for each patient. The energy intake was monitored and consistent throughout the study. Patients grew and gained weight as expected. Parents were trained to take blood spots, and their technique was checked. The laboratory rejected for analysis any blood spots that were small or of poor quality.

There were several limitations that need consideration. Metabolic control is influenced by dietary intake but also by factors such as catabolism associated with illness. Fortunately, this cohort was generally well throughout the study, but minor illnesses could have increased some of the blood phenylalanine above therapeutic target range. Diet diaries have a degree of uncertainty, as some food and drink intake may be unreported, although many of the children ate a limited range of meal choices, choosing similar foods from day to day. Genetic mutation analysis was not completed at the time of the study, so we were not able to analyze the possible influence of specific genotypes on natural protein tolerance and different responses to the interventions. For ethical reasons and good clinical practice, we minimized the length of time blood phenylalanine was above the upper target range. Therefore, mean blood phenylalanine in Phase D and E must be interpreted carefully, as it does not show the full effect of the additional phenylalanine intake from milk sources.

## 5. Conclusions

This evidence supports the PKU European Guidelines upper cutoff value for the phenylalanine content for use of fruits and vegetables without measurement in a phenylalanine-restricted diet. All patients with PKU were able to tolerate an extra 50 mg/day but not 100 mg/day of phenylalanine from fruits and vegetables. Tolerance of the ‘free use’ of fruits and vegetables containing phenylalanine 76–100 mg/100 g depended on inter-patient variability, and they cannot be recommended for patients without controlled measurement of intake in PKU. Considering patients responded differently to the same intervention; it was challenging to calculate the length of time necessary to anticipate the impact on blood phenylalanine control of any increase in dietary phenylalanine intake when establishing maximum natural protein tolerance. In our study, there was a suggestion of more rapid increase with less stability of blood phenylalanine with milk protein compared with fruit and vegetable protein. More studies are necessary to identify the different impact on blood phenylalanine of different protein sources in patients with PKU.

## Figures and Tables

**Table 1:** Phenylalanine content (mg/100 g), and 50 mg phenylalanine exchange portion sizes for fruits and vegetables and milk sources given in the intervention period of this study.

Food	Phenylalanine Content mg/100 g *	Portion Size Weight for 1 Phenylalanine Exchange (50 mg)
***Beansprouts***	92 mg	60 g
***Broccoli***	76 mg	60 g
***Cauliflower***	89 mg	60 g
***Yam***	97 mg	60 g
***Sugar snap peas***	75–88 mg	60 g
***Mange tout***	66–93 mg	60 g
***Bamboo Shoots***	83 mg	60 g
***Brussel sprouts***	83 mg	60 g
***Figs***	83 mg	60 g
***Milk***	180 mg	30 mL
***Yoghurt***	Phenylalanine content calculated from protein content (g/100 g) of yoghurt on packaging. Assumed 1 g protein = 50 mg phenylalanine	
***Milk based ice cream***	Phenylalanine content calculated from protein content (g/100 g) of ice cream on packaging. Estimated that the weight of ice cream that provided 1 g protein = 50 mg phenylalanine	

* [17].

**Table 2:** Patient characteristics and dietary prescription at the start of the study.

Subject Number	Age (Years)	Sex	Phenotype	Randomization (Starting Intervention)	Prescribed Daily Number of 1 g Protein/50 mg Phenylalanine Exchanges	Protein Equivalent Amount (g/day) from Protein Substitute	Protein Substitute Brand, Size and Manufacturer
1	9	Female	Classical PKU	Fruit/vegetables	4	65	PKU Air 15^®^ Vit
							(45 g PE)
							PKU Air 20^®^ Vit
							(20 g PE)
2	7	Male	Classical PKU	Milk products	6.5	60	PKU Express 15^®^ Vit
							(45 g PE)
							PKU Air 15^®^ Vit
							(15 g PE)
3	12	Male	Mild PKU	Fruit/vegetables	25	60	PKU Sphere 20^®^ Vit
							(60 g PE)
4	12	Female	Mild PKU	Fruit/vegetables	14	60	PKU Sphere 20^®^ Vit
							(60 g PE)
5	9	Female	Classical PKU	Milk products	6	60	PKU Sphere 20^®^ Vit
							(60 g PE)
6	12	Male	Classical PKU	Milk products	7.5	80	PKU Sphere 20^®^ Vit
							(60 g PE)
							PKU Cooler 20^®^ Vit
							(20 g PE)
7	12	Female	Classical PKU	Milk products	4.5	80	PKU Sphere 20^®^ Vit
							(80 g PE)
8	8	Male	Classical PKU	Fruit/vegetables	3	80	PKU Sphere 20^®^ Vit
							(60 g PE)
							PKU Cooler 20^®^ Vit
							(20 g PE)
9	6	Female	Classical PKU	Milk products	5.5	60	PKU Cooler 15^®^ Vit
							(60 g PE)
10	11	Female	Classical PKU	Fruit/vegetables	4	60	PKU Air 20^®^ Vit
							(40 g PE)
							PKU Sphere 20^®^ Vit
							(20 g PE)
11	11	Female	Classical PKU	Milk products	6	60	PKU Lophlex LQ 10^®^ Nu
							(60 g PE)
12	11	Female	Classical PKU	Fruit/vegetables	5	60	PKU Air 20^®^ Vit
							(40 g PE)
							PKU Sphere 20^®^ Vit
							(20 g PE)
13	10	Female	Classical PKU	Milk products	5.5	60	PKU Lophlex LQ 20^®^ Nu
							(60 g PE)
14	12	Female	Classical PKU	Fruit/vegetables	6	70	PKU Cooler 20^®^ Vit
							(60 g PE)
							PKU Cooler 10^®^ Vit
							(10 g PE)
15	8	Male	Classical PKU	Milk products	4	60	PKU Air 20^®^ Vit
							(40 g PE)
							PKU Sphere 20^®^ Vit
							(20 g PE)
16	9	Female	Classical PKU	Fruit/vegetables	6	60	PKU Air 20^®^ Vit
							(60 g PE)

Abbreviations: PKU, phenylketonuria; PE, protein equivalent; Vit, Vitaflo (Liverpool, UK); Nu, Nutricia (Trowbridge, UK).

**Table 3:** Tolerance of additional phenylalanine in children with PKU from either fruits/vegetables or milk protein.

	Dietary Intervention			
	*Phase B:* An Additional 50 mg/day Phenylalanine from Fruits and Vegetables (Phenylalanine Containing 76–100 mg /100 g)	*Phase C:* An Additional 100 mg/day Phenylalanine from Fruits and Vegetables (Phenylalanine containing 76–100 mg /100 g)	*Phase D:* An Additional 50 mg/day Phenylalanine from Milk Protein	*Phase E:* An Additional 100 mg/day Phenylalanine from Milk Protein
% subjects that maintained blood phenylalanine control * within target range over 4 weeks	100% (*n =* 16/16)	69% (*n* = 11/16)	50% (*n =* 8/16)	31% (*n =* 5/16)

* Loss of metabolic control: 4/6 consecutive blood phenylalanine levels > 360 μmol/L.

**Table 4:** Number of weeks taken for blood phenylalanine levels to elevate above upper target therapeutic range with each intervention.

Number of Weeks Need for Blood Phenylalanine Levels Above Target Range																
	Subject 1	Subject 2	Subject 3	Subject 4	Subject 5	Subject 6	Subject 7	Subject 8	Subject 9	Subject 10	Subject 11	Subject 12	Subject 13	Subject 14	Subject 15	Subject 16
**Milk protein** **intervention**	8 weeks	N/A	N/A	2 weeks	4 weeks	3 weeks	2 weeks	2 weeks	2 weeks	4 weeks	N/A	4 weeks	N/A	6 weeks	N/A	7 weeks
**Fruits and vegetables protein intervention**	N/A	6 weeks	N/A	8 weeks	N/A	N/A	N/A	N/A	8 weeks	N/A	N/A	N/A	N/A	N/A	6 weeks	8 weeks

Abbreviation: N/A, not applicable as Phe levels did not elevate >360 μmol/L.

**Table 5:** Mean change in blood phenylalanine from baseline by week and study intervention.

Change in Blood Phenylalanine from Baseline by Week and Study Intervention																
	50 mg/day Phenylalanine from Fruit and Vegetable Protein				100 mg/day Phenylalanine from Fruit and Vegetable Protein				50 mg/day Phenylalanine from Milk Protein				100 mg/day Phenylalanine from Milk Protein			
	Week 1	Week 2	Week 3	Week 4	Week 1	Week 2	Week 3	Week 4	Week 1	Week 2	Week 3	Week 4	Week 1	Week 2	Week 3	Week 4
**Mean change of blood phenylalanine from baseline** **(µmol/L)**	4	−7	−29	76	24	18	2	35	20	41	60	131	6	10	98	154
**Number of** **subjects**	16	16	16	16	16	16	14	14	16	16	12	11	8	8	7	6
***p*-value**	0.924	0.788	0.492	0.082	0.496	0.617	0.955	0.364	0.59	0.292	0.231	0.062	0.893	0.846	0.208	0.049

**Table 6:** Phenylalanine intake (mg/day) in each phase of the study.

Dietary Intake in Each Study Phase					
	Phase A (No Intervention)	Phase B (An Additional 50 mg/day Phenylalanine from Fruits and Vegetables)	Phase C (An Additional 100 mg/day Phenylalanine from Fruits and Vegetables)	Phase D (An Additional 50 mg/day Phenylalanine from Milk Protein)	Phase E (An Additional 100 mg/day Phenylalanine from Milk Protein)
**Median phenylalanine intake (range) in mg/day**	500 (range: 325–1525)	588 (range: 450–1650)	638 (range: 400–1725)	563 (range: 475–1650)	625 (range: 600–1725)
**Number of patients completing 4 weeks of intervention**	16	16	14	11	6

## Data Availability

Not applicable.

## Supplementary Materials

The following supporting information can be downloaded at: https://www.mdpi.com/article/10.3390/nu14204268/s1, Table S1. Natural protein intake and intervention in each week of the study. Table S2. Mean change between milk protein intervention compared with fruit and vegetables protein.
