# Supplementary material for: Impact of Fruit and Vegetable Protein vs. Milk Protein on Metabolic Control of Children with Phenylketonuria: A Randomized Crossover Controlled Trial
Source: Nutrients. 2022 Oct 13;14(20):4268. doi: 10.3390/nu14204268 (PMC9611310; doi:10.3390/nu14204268)
Supplement: Supplementary file 1 [file nutrients-14-04268-s001.zip › nutrients-1955490-supplementary.pdf]

nutrients

MDPI

Supplementary Table S1. Natural protein intake and intervention in each week of the study.

Dietary Intake During Each Week of the Study				
	**Intervention**	**Number of Subjects Who Stopped Each Arm of The Study Due to High Blood Phenylalanine Levels**	**Median Daily Exchanges Prescribed (Range)**	**Sources Used for Extra Phe (Number of Times Food was Used During 3-day Diet Diary)**
**Phase A week 1 (*n* = 16)**	**Baseline**	n/a	5.75 (3–25)	n/a
**Phase A week 2 (*n* = 16)**		n/a	5.75 (3–25)	n/a
**Phase A week 3 (*n* = 16)**		n/a	5.75 (3–25)	n/a
**Phase A week 4 (*n* = 16)**		n/a	5.75 (3–25)	n/a
**Phase B week 1 (*n* = 16)**	**Extra 50 mg of Phenylalanine from fruit/vegetables**	0	6.75 (4–26)	Broccoli (37); Cauliflower (5); Mange tout (2); Sugar snap peas (1); Beansprouts (1)
**Phase B week 2 (*n* = 16)**		0	6.75 (4–26)	Broccoli (40); Cauliflower (4); Beansprouts (1); Figs (1)
**Phase B week 3 (*n* = 16)**		0	6.75 (4–26)	Broccoli (37); Figs (2); Cauliflower (4); Sugar snap peas (2); Beansprouts (1)
**Phase B week 4 (*n* = 16)**		0	6.75 (4–26)	Broccoli (38); Figs (1); Sugar snap peas (3); Cauliflower (3); Beansprouts (1)
**Phase C week 1 (*n* = 16)**	**Extra 100 mg of Phenylalanine from fruit/vegetables**	0	7.75 (5–27)	Broccoli (40); Cauliflower (3); Figs (4); Sugar snap peas (3); Beansprouts (1)
**Phase C week 2 (*n* = 16)**		2	7.75 (5–27)	Broccoli (37); Cauliflower (6); Figs (3); Sugar snap peas (3); Bamboo shoots (1)
**Phase C week 3 (*n* = 14)**		0	7.75 (5–27)	Broccoli (31); Cauliflower (4); Figs (4); Sugar snap peas (2); Green beans (1); Brussel sprouts (2)
**Phase C week 4 (*n* = 14)**		1	7.75 (5–27)	Broccoli (29); Cauliflower (6); Figs (4); Sugar snap peas (3)
**Phase D week 1 (*n* = 16)**	**Extra 50 mg of Phenylalanine from milk protein**	0	6.75 (4–26)	Ice cream (30); Milk (10); Yoghurt (7)
**Phase D week 2 (*n* = 16)**		3	6.75 (4–26)	Ice cream (31); Milk (8); Yoghurt (7)
**Phase D week 3 (*n* = 12)**		1	6.75 (4–26)	Ice cream (28); Milk (3); Yoghurt (4)
**Phase D week 4 (*n* = 11)**		4	6.75 (4–26)	Ice cream (32)
**Phase E week 1 (*n* = 8)**	**Extra 100 mg of Phenylalanine from milk protein**	0	7.75 (5–27)	Ice cream (17); Yoghurt (5)
**Phase E week 2 (*n* = 8)**		0	7.75 (5–27)	Ice cream (17); Yoghurt (5)
**Phase E week 3 (*n* = 7)**		2	7.75 (5–27)	Ice cream (17); Yoghurt (5)
**Phase E**		1	7.75	Ice cream (10); Yoghurt (4)

**week 4 (*n* = 6)**	(5–27)

N/A: not applicable.

Supplementary Table S2. Mean change between milk protein intervention compared with fruit and vegetables protein.

**Mean Change in Macronutrients Intake between Interventions**					
	**Energy (kcal)**	**Energy (% of Energy Requirements)**	**Carbohydrate (% of Total Energy Intake)**	**Fat (% of Total Energy Intake)**	**Protein (% of Total Energy Intake)**
**Mean change of milk protein vs fruit and vegetables protein**	68	3.3	−0.03	0.3	−0.3
***p*-value**	0.188	0.102	0.961	0.64	0.329
